# A dichoptic feedback-based oculomotor training method to manipulate interocular alignment

**DOI:** 10.1038/s41598-020-72561-y

**Published:** 2020-09-24

**Authors:** Andrea Caoli, Silvio P. Sabatini, Agostino Gibaldi, Guido Maiello, Anna Kosovicheva, Peter Bex

**Affiliations:** 1 Department of Psychology, Northeastern University, Boston, MA 02115 USA; 2 Department of Informatics, Bioengineering, Robotics and System Engineering-DIBRIS, University of Genoa, Via all’Opera Pia 13, 16145 Genoa, Italy; 3 School of Optometry and Vision Science, University of California at Berkeley, Berkeley, CA 94720 USA; 4 Department of Experimental Psychology, Justus Liebig University Giessen, 35394 Giessen, Germany

**Keywords:** Psychology, Ocular motility disorders, Vision disorders, Oculomotor system, Visual system

## Abstract

Strabismus is a prevalent impairment of binocular alignment that is associated with a spectrum of perceptual deficits and social disadvantages. Current treatments for strabismus involve ocular alignment through surgical or optical methods and may include vision therapy exercises. In the present study, we explore the potential of real-time dichoptic visual feedback that may be used to quantify and manipulate interocular alignment. A gaze-contingent ring was presented independently to each eye of 11 normally-sighted observers as they fixated a target dot presented only to their dominant eye. Their task was to center the rings within 2° of the target for at least 1 s, with feedback provided by the sizes of the rings. By offsetting the ring in the non-dominant eye temporally or nasally, this task required convergence or divergence, respectively, of the non-dominant eye. Eight of 11 observers attained 5° asymmetric convergence and 3 of 11 attained 3° asymmetric divergence. The results suggest that real-time gaze-contingent feedback may be used to quantify and transiently simulate strabismus and holds promise as a method to augment existing therapies for oculomotor alignment disorders.

## Introduction

Strabismus is an impairment of ocular alignment that has a prevalence of around 3–5%^1–6^**.** One or both eyes may deviate in (*esotropia*) or out (*exotropia*), or less frequently, up (*hypertropia*), down (*hypotropia*) or rotationally (*cyclodeviation*). The angle of deviation may be *constant* or *intermittent,* with angles below 5° termed *microtropia*, and may be present in all (*comitant*) or a subset of (*noncomitant*) gaze directions (for review see^7^). Strabismus is associated with a range of sensory and motor deficits (for review see^8^). If strabismus occurs during critical periods of development, there is a significant risk of amblyopia (for review see^9^). Strabismic amblyopia is associated with deficits in visual acuity^10,11^, contrast sensitivity^12,13^, and stereoacuity (for reviews see^14,15^), as well as diplopia^16^, interocular suppression^17^, asthenopia^18^, and abnormal head posture^19^ and gait^20^. Many of these binocular sensory deficits persist for people with strabismus whose amblyopic monocular visual acuity deficit has been successfully treated^21^. Furthermore, people with strabismus may suffer from decreased quality of life, including reductions in self-esteem and self-confidence^22–28^, and diminished employment^29^, academic^30,31^ and sports opportunities^32^ (for reviews see^32,33^).

The primary means of diagnosing and monitoring strabismus is the cover test, which requires an examiner to detect a refixation movement of a deviated eye when the dominant eye is covered^34^. Although this provides an objective measurement of alignment, cover test results also depend on experience of the examiner^35^ and age of the patient^36^. For example, cover test measurements performed on the same individual by different examiners vary by up to 6–12 prism diopters in primary position, depending on the magnitude of the deviation^37^. This corresponds to 3.4°–6.8° of visual angle which is considerably lower than the accuracy and precision of most eye trackers. More recently, cover testing has been automated with dichoptic displays to occlude each eye, paired with eye trackers that quantify refixation movements of the deviating eye^38–41^. Alternatively, imaging methods such as the Hirschberg test^42^ detect interocular decentrations of the line of sight and a cellphone-based version of this test is now available^43^. These methods are primarily used for the measurement of ocular deviation in primary, straight-ahead gaze. Estimates of ocular deviation at non-primary directions of gaze can be obtained with time-consuming binocular alignment methods, including the synoptophore (invented by Walter Green, made by Clement Clarke, 1931–1940 see: collection.sciencemuseum.org.uk), Hess^44^, Lancaster^45^, Lees^46^ and Harms Tangent^47^ screens. In contrast, ocular deviations can be measured at multiple gaze directions in 90 s with eye tracking methods and automated occlusion with stereoscopic displays^38,39^.

Strabismus may be corrected by surgery, which leads to improvements in oculomotor control^48,49^ and quality of life indicators^24,28,29,50–55^. However, while some studies have reported immediate benefits in stereoacuity following ocular alignment surgery^56–60^, others have found little or no benefit^61–63^ and there may be adverse impact on postural stability^64,65^. This suggests that perceptual therapy may be required in addition to surgery and that superior outcomes may be observed in patients for whom stereopsis is present before surgery^66^ (for review see^3^). A surgical outcome is considered successful where 10 diopters or less of horizontal deviation, or 4 diopters of vertical deviation are initially obtained, but this may not be retained^67^ and additional corrective surgery is required in 20–50% cases^68^. Furthermore, surgical complications are frequent^69^ and over or under-correction of ocular deviation is common^70^.

Alternatively, strabismus may be corrected non-surgically, for example, with prism lenses that alter the light path by an angle opposite to the angle of deviation. Although this method is non-invasive, it does not resolve the problem in noncomitant strabismus^71^ and prism use may alter body posture^64^. Similarly, an eye tracker-enabled dichoptic display has been used to shift images in real time to compensate for deviation and this method has been shown to facilitate fusion^72^. Vision therapy for correction of small angle deviation includes ocular motility exercises, such as Pencil Push Ups, Brock Strings^73^ and Barrel Cards. In these training tasks, the patient tracks a target that moves in depth, or alternates fixation among targets at different depths, in order to promote ocular motility and single vision (for review see^74,75^). Vision Therapy may be combined with surgery, which together can promote long term outcomes (for a review see^76^). However, the efficacy of Vision Therapy remains controversial (for reviews see^71,77–83^). Considerably less attention has been directed in recent years to the use of feedback for the treatment of strabismus (for review see^84,85^). In most cases, ocular alignment feedback was provided by auditory tones with successful case study reports^86–88^.

In healthy observers, many groups have demonstrated that eye movements can be modified based on sensory feedback. In the classic *double step* paradigm^89^, a target is shifted during a saccade, and therefore poorly visible to the observer^90,91^, causing a mismatch between the expected and actual foveal endpoint. After many trials, the gain of the oculomotor system recalibrates to correct the error (for review see^92^). We recently modified this paradigm with dichoptic feedback to deliver double steps in different directions in each eye^93^. With this paradigm, we transiently induced and reversed ocular misalignment over the course of approximately 150 saccadic eye movements. In follow up research, we showed that the apparent locations of targets estimated by the perceptual system were affected by these adaptive changes in saccade amplitude^94^. In related research, we employed real-time visual feedback to train healthy observers to change their gaze behavior to use eccentric fixation^95^. Observers learned to control the on-screen position of a gaze-contingent ring with their eye movements. Feedback was provided by the size of the ring, which decreased when the eccentric fixation target was within the ring and decreased when the target was outside the ring. Observers learned to control the position of the ring and fixation stability significantly improved over 4 training blocks. Furthermore, these improvements in the accuracy and precision of eccentric fixation were partially retained over a week.

In the present study, we extend these feedback-based approaches in a proof-of-concept study in normally-sighted observers to manipulate interocular alignment. We use a standard stereoscopic display and eye tracker to control stimuli independently for each eye. A static fixation target is presented only to the observer’s dominant eye (assessed with a contrast balance paradigm^17^), and independent gaze-contingent rings are presented to both the observer’s dominant and non-dominant eyes. The observer’s task is to make self-initiated asymmetric vergence eye movements to control the positions of the rings so that they are both centered on the fixation target. Feedback is provided by the size of the rings, which change in real-time and decrease when the target is within the rings or increase if the target is outside the rings. In Experiment 1, the rings were centered on the fovea of each eye, which provided training on the feedback method. In Experiment 2 the ring presented to the non-dominant eye (NDE) was offset temporally, which required the observer to deviate the NDE inwards (asymmetric convergence) to center the rings on the target. In Experiment 3 the ring presented to the NDE was offset nasally, which required outward deviation of the NDE (asymmetric divergence) to center the rings on the target. The goal of Experiments 2 and 3 was an exploratory investigation of whether feedback could be used to train transient asymmetric convergent or divergent fixation. In addition, given the consequences of strabismus for binocular sensory processing, we tested whether this transiently-induced misalignment was associated with changes in sensory eye dominance.

## Methods

### Participants

Eleven participants (seven female, four male) with self-identified normal or corrected-to-normal vision were recruited from the senior authors’ laboratory and from the undergraduate student population at Northeastern University. Undergraduates received course credit towards the completion of their Introductory Psychology course in exchange for their participation. All were naive to the purposes of the study at intake and indicated their willingness to participate by signing an informed consent document associated with a protocol approved by the University Ethics Board. The Northeastern University Ethics Board evaluated the protocol and confirmed that this research adhered to the tenets of the Declaration of Helsinki.

### Apparatus

Eye movements were recorded at a sampling rate of 1000 Hz with an SR Research Eyelink 1000 infrared eye tracking system, used in conjunction with the Eyelink Toolbox for Matlab^96^. Observers were calibrated with a standard 9-point calibration procedure^97^ at the beginning of each block of trials while viewing the targets binocularly through the same shutter glasses used in the experiment.

Stimuli were presented on a gamma-corrected 27″ BenQ XL2720Z LCD monitor controlled by a Dell XPS 8300 desktop computer with a Quadro FX 4600 graphics card. The experiment was programmed in Matlab (The Mathworks, Inc., Natick, MA) using the Psychophysics Toolbox Version 3^98–100^. Display resolution was set to 1920 × 1080 at 120 Hz with a mean luminance of 25 cd/m^2^ through the shutter glasses. Color bit stealing was used to generate 10.8 bits of greyscale resolution^101^. Observers viewed the display binocularly through LCD active shutter glasses synchronized to the refresh of the monitor (NVidia 3D Vision; 60 Hz monocular refresh). The cross talk of the dichoptic system was 1% measured with a Spectrascan 6500 photometer. Eye movement data were successfully recorded through the shutter glasses, as in previous work^93,102–104^. Observers were seated at a viewing distance of 60 cm from the display, with head position stabilized using a chinrest. At this distance, the display subtended 52.9° horizontally and 31.2° vertically.

### Procedure

**Figure 1:**
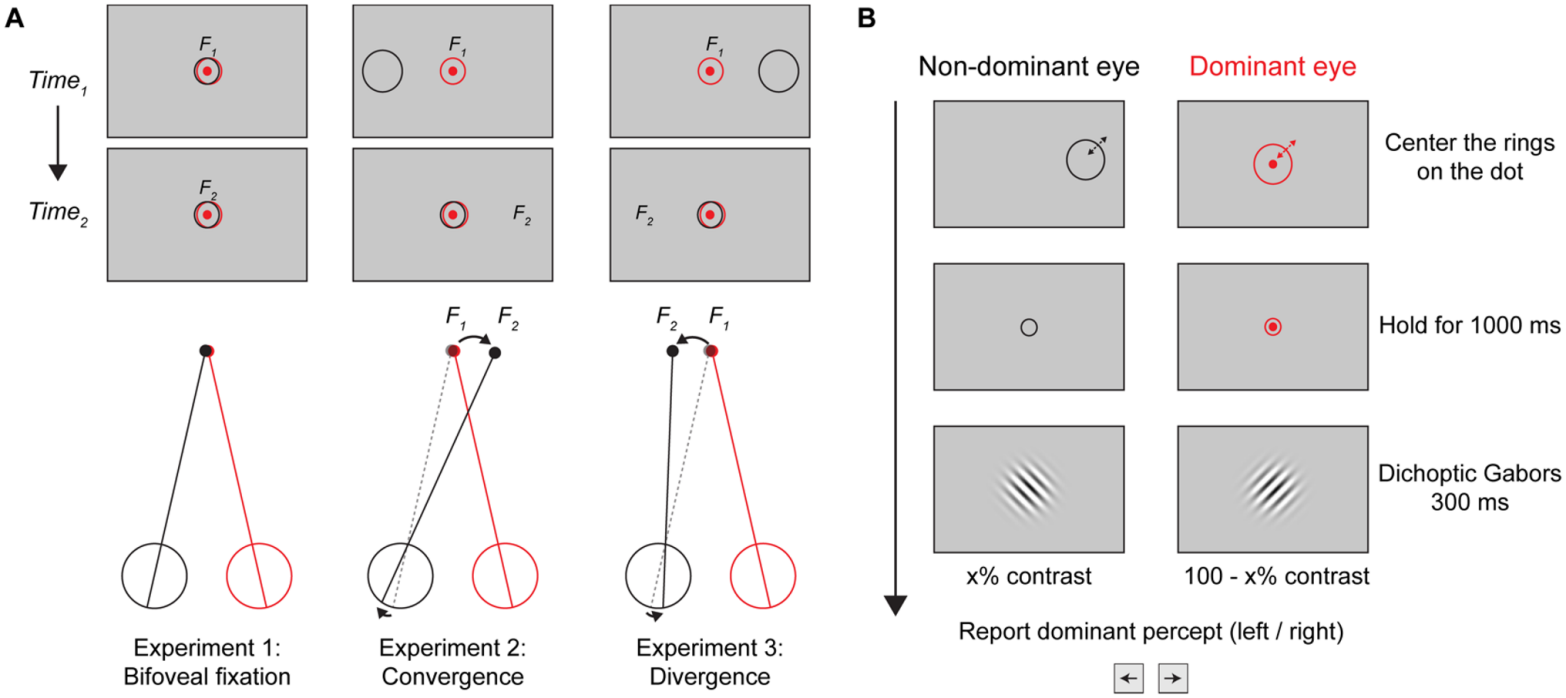
Illustration of the training procedure with dichoptic feedback. (**A**) Stereoscopic shutter glasses were used to control the presentation of different images to the two eyes while eye alignment was manipulated in Experiments 1–3 (from left to right): bifoveal fixation, convergence, and divergence. The non-dominant and dominant eyes are shown in black and red (left and right eyes, respectively). In the bifoveal fixation experiment, observers were instructed to maintain fixation centrally with both eyes. In the other two experiments, observers maintained fixation with the dominant eye on the target, while shifting the gaze direction of the nondominant eye either nasally (Exp 2: convergence) or temporally (Exp 3: divergence). A stationary target dot was shown only to the dominant eye (shown in red), and gaze-contingent feedback was provided by expanding/contracting rings shown independently to the two eyes (red and black). The observer’s task was to move their eyes so that the rings were centered on the target dot. The fixation location of the nondominant eye at two different time points (time_1_ and time_2_) is indicated by F_1_ and F_2_ (not visible to subjects). At time 1, the eyes are aligned, and at time 2, the observer has completed an asymmetric vergence movement (Experiments 2 and 3), to move the gaze-contingent ring over the stationary target. In Experiments 2 and 3, a constant horizontal offset (5° in Experiment 2 and 3° in Experiment 2) was added to the ring in the non-dominant eye, such that the rings would be centered on the target when the correct gaze positions were held. Each ring decreased in size when it overlapped with the target dot, and increased when it was outside the target dot. The procedure for each trial of the Ocular Alignment task is shown in (**B**), using an example from Experiment 3. The ring and dot in the dominant eye were displayed in white to observers (shown in red here for visibility). Observers were instructed to center the two rings over the dot and hold this posture for 1000 ms. Immediately afterward, sensory eye dominance was measured with a pair of dichoptic Gabors with variable contrast (see “Methods”) and different orientations (− 45° and + 45°). Observers then reported the orientation of the dominant percept with a key press.

Each observer first completed a pretest measurement of sensory eye dominance, which consisted of 80 trials of only the Eye Dominance procedure described below. This task was completed to quantify binocularity instead of a stereoacuity task. Observers then completed 1 to 3 experiments of an Ocular Alignment task (40 trials each), according to their ability to converge and/or diverge their eyes. As shown in Fig. 1B, each trial in Experiments 1–3 consisted of a gaze-contingent feedback procedure, followed by the same eye dominance task used in the pretest. All observers attempted each experiment initially. If the observer was unable to reliably center the rings on the target (see “Procedure”) over the course of approximately 10 trials (with up to 1–2 min attempts for each), the experiment was aborted. A total of 11, 8, and 3 participants were able to complete Experiments 1, 2, and 3, respectively (see “Results” for details). Aside from the Bifoveal task (Experiment 1), which provided all observers with training in the use of real-time feedback, observers were not given additional prior training or practice, as our goal was to examine how observers learned to perform these tasks. In all tasks, in order to train asymmetric vergence across different gaze directions, the location of the target was randomly selected on each trial from nine locations, corresponding to a 3 × 3 invisible grid within the central 15 degrees of the display.

#### Eye dominance

Sensory eye dominance was measured in a pretest (80 trials) and during all experiments (40 trials) with a binocular balance paradigm^17,105^. The results of the pretest were used to estimate the dominant eye as the eye reported when the stimulus contrast in each eye was equal. In this task, the stereoscopic display was used to present a different Gabor patch, *G*, to each eye:

$${G}{\left(x,y\right)}=\exp\left({-\frac{{x}^{2}+{y}^{2}}{2{\sigma }^{2}}}\right)\times\cos\left[2\pi f \times \left(x\cos\theta +y\sin\theta\right) +\varphi \right]$$

where the standard deviation of the circular Gaussian envelope, *σ*, was 1.7°, the grating’s orientation, *θ*, was − 45° in one eye and + 45° in the other eye, spatial frequency, *f*, was 4 c/deg and phase, *φ*, was 0° at the center of the image. The large size of the Gabors ensured significant dichoptic overlap, even when the observer’s eyes were convergent or divergent. The Michelson contrast of the Gabor in the dominant eye was equal to 1 minus the contrast of the Gabor in the non-dominant eye, such that the contrast across both eyes summed to 1. The relative contrast of the Gabors was under the control of an adaptive QUEST staircase^106^ that converged on a contrast producing 50% responses for the stimulus presented to the left and right eye, referred to here as the interocular balance contrast. The starting contrast of the dominant eye Gabor was randomly drawn from a uniform distribution in the interval 0.4–0.6. The observer’s task was to indicate the orientation of the dominant percept in the overlapping region of the two Gabors by pressing the one of two arrow keys: left for counter-clockwise (− 45°) or right for clockwise (45°).

The Gabors were presented for 300 ms, with abrupt onset and offset. This duration was determined from pilot trials to ensure that the gaze posture established in the ocular alignment task was maintained during the eye dominance task. Our results confirm this in the main experiment.

Observers' responses from the Eye Dominance pretest measurement were used to determine sensory eye dominance for the purposes of stimulus presentation in the Ocular Alignment task. The balance contrast was estimated from the QUEST data, based on the mean of the posterior probability density function of the threshold after 80 pretest trials and the dominance was assigned to the eye that required < 0.5 contrast.

#### Ocular alignment

A dichoptic display was used to present a fixation target only to the observer’s dominant eye and two gaze-contingent feedback rings (one drawn independently per eye). The fixation target was a white (50 cd/m^2^ through the shutter glasses) dot that subtended 1.6° diameter.

The procedure for each trial is shown in Fig. 1B. A white gaze-contingent feedback ring (line width 1.7 arcmin) was presented at the foveal gaze direction of the dominant eye, estimated by the eye tracker. A second, black (≈ 0 cd/m^2^) gaze-contingent feedback ring was presented at a location relative to the foveal direction of gaze of the non-dominant eye, with a constant horizontal offset that varied between conditions (see descriptions below for each experiment). The rings were the same sizes in the two eyes at all times, with an initial size of 1.38° and were continuously and independently updated every 16.7 ms based on relative position of the target dot and the feedback ring. The diameter of both rings decreased by 1.7 arcmin per frame (1 pixel @60 Hz per eye, equivalent to 1.7°/s) if the dot was inside both rings or increased by 1.7 arcmin per frame if it was outside either ring, up to a maximum size of 4.13°. This feedback ensured that the task was possible for observers of any ability level, since the ring could increase in size (in principle to cover the whole display if needed, although this could mean that very large rings may be less visible than smaller rings). Feedback also continuously promoted alignment of the ring on the dot, regardless of the ring size. As shown in Fig. 1B, on each trial, the observer’s task was to move their eyes to control the position of the gaze-contingent rings in an attempt to center both rings on the dot. At any point during a trial, observers were permitted to abort the trial for any reason if they were unable to complete the task. Once the observer held the centers of both rings within 2° of the center of the dot for at least 1 s, the target dot and rings were replaced with the eye dominance test stimuli (dichoptic Gabors) for 300 ms, centered on the location of the target dot. After pressing a key to indicate the orientation of the dominant percept (left or right tilt), the observer was allowed unlimited time to rest before pressing the spacebar to initiate the next trial. This process of alignment and dominance test was repeated until observers successfully completed 40 trials for each experiment.

The location of the ring in the non-dominant eye relative to the registered gaze position from the eye tracker was adjusted in different experiments to manipulate ocular alignment (Fig. 1).

##### Experiment 1—bifoveal fixation

The gaze-contingent ring was centered on the fovea of the non-dominant eye, so that the foveae of both eyes were directed at the location of the target dot.

##### Experiment 2—asymmetric convergence

The gaze-contingent ring was centered 5° (8.75Δ) in the temporal retina of the non-dominant eye. In this case, the dominant eye was directed at the target dot, but the non-dominant eye must be directed 5° nasally in order to center the ring on the dot. This arrangement simulates esotropia^107^.

##### Experiment 3—asymmetric divergence

The gaze-contingent ring was centered 3° (5.24Δ) in the nasal retina of the non-dominant eye. In this case, the dominant eye was directed at the target dot, but the non-dominant eye must be directed 3° temporally in order to center the ring on the dot. This arrangement simulates exotropia^107^. In pilot studies, we attempted to match the 5° employed in Experiment 2 for convergence, however, neither of the pilot observers were able to perform this task.

### Gaze data post-processing

Eye position data were collected at 1000 Hz and analyzed offline with Matlab. Noise artifacts were reduced using Eyelink software, which applied a heuristic filtering algorithm to the raw gaze position samples^97^. In addition, data that were lost during blinks etc. were interpolated with Matlab’s function *pchip()*^108^*,* a method that employs shape-preserving piecewise cubic spline interpolation. After interpolation, outlier data points were detected by Matlab’s function *movmedian()*, where outliers are defined as elements more than three local scaled Median Absolute Deviation (MAD)^109,110^ from the local median over a window length specified by the length of all the outlier data times two. For a random variable vector A, composed of N scalar observations, MAD is defined as:

$$MAD = median \left( {\left| {A_{i} - median\left( A \right)} \right|} \right)\quad for\;\; i = 1,2, \ldots , N.$$

Outlier data were replaced with the local median value. Then, the prebuilt function filled with the upper threshold value for elements larger than the upper threshold determined by *movmedian()*. We used a Threshold Factor equal to 3/2, for detecting outlier data.

For each trial, we extracted and analyzed gaze positions during time points ranging from − 2000 to + 2000 ms relative to the onset of the dichoptic Gabors. We note that on some trials, observers were able to complete the task and initiate the next trial quickly, which resulted in a small degree of temporal overlap in the gaze position traces analyzed across trials (e.g., the last 1000 ms of the trace from trial 1 could correspond to the same time points as the first 1000 ms of the trace from trial 2). Overall, the proportion of trials in which the length of overlap was more than 1000 ms was very low (8.6%, 1.3%, and 4.3% of all trials for Experiments 1, 2, and 3, respectively), and the results are very similar when overlapping data points are removed from the analysis (see Figure S1 in the Supplemental Materials).

## Results

### Experiment 1—bifoveal fixation

**Figure 2:**
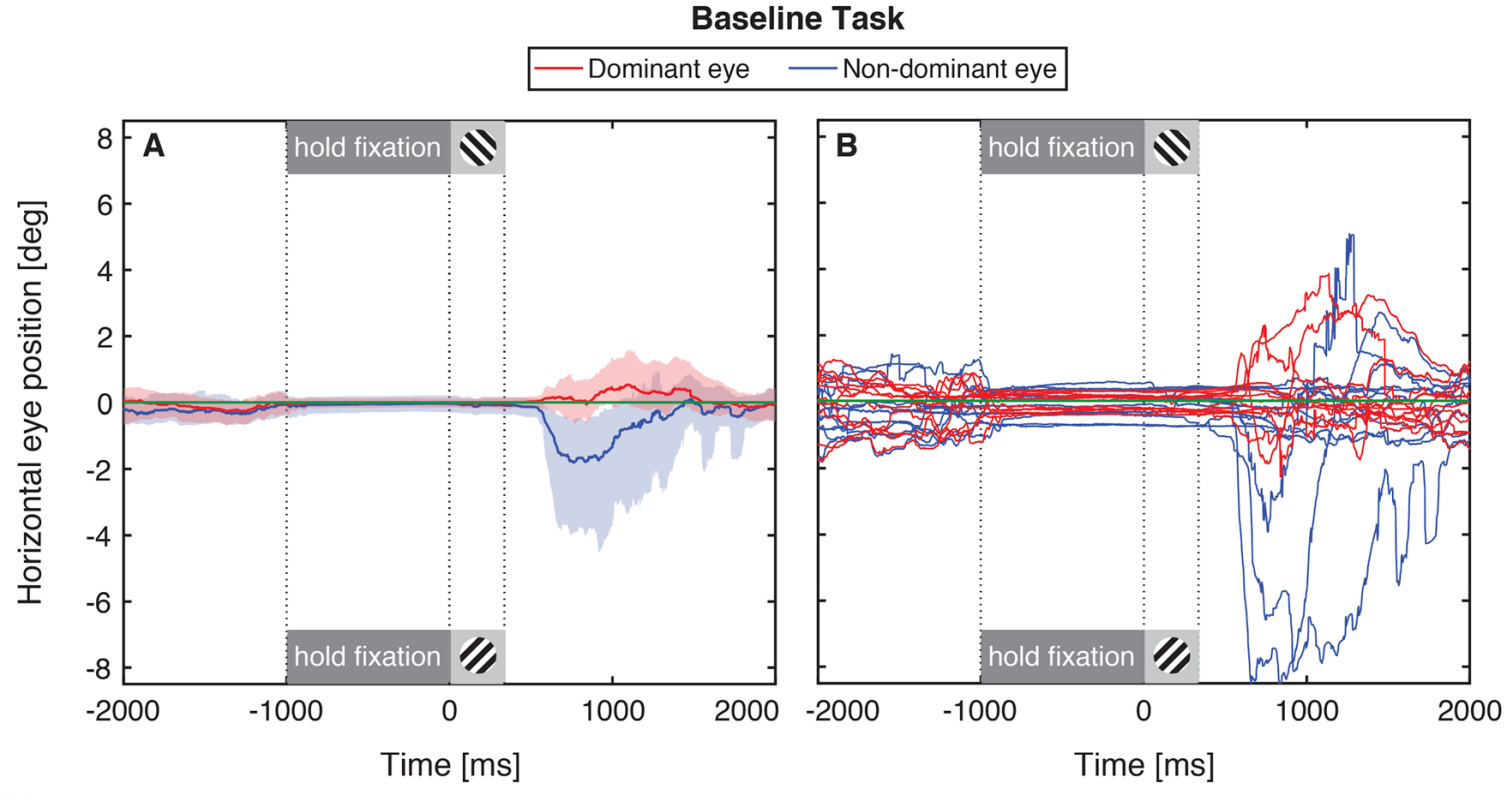
Horizontal gaze during bifoveal fixation. The horizontal position of the dominant (red traces) and non-dominant (blue traces) eyes of 11 observers in Experiment 1. Gaze positions are shown relative to the intended final gaze direction in the non-dominant eye (F_2_ in Fig. 1), and the green line (at 0°) indicates the location of the target dot in the dominant eye. Time points are shown from − 2000 to + 2000 ms, relative to the onset time of the dichoptic Gabors (0 ms). The mean data for 40 trials for 11 observers are plotted in (**A**) and shaded regions show bootstrapped 95% confidence intervals. The mean data for 40 trials for each individual observer are shown in (**B**). Time points at 0 ms identify the end of the alignment task (indicating that observers had maintained the required fixation pattern for at least 1 s) and the start of the eye dominance task. The dark gray shaded box from − 1000 to 0 ms identifies the interval in which observers maintained successful ocular alignment, with times < − 1000 ms showing eye position while observers were attempting to center the ring on the target. The light gray shaded box from 0 to 300 ms indicates the interval of the eye dominance test (with Gabors of opposite orientations shown to the two eyes). Observers were able to move their eyes freely for times > 300 ms. See text for details.

Observers were required to maintain the foveae of both eyes on the fixation target for at least 1 s before completing the eye dominance test. Each eye controlled the position of a gaze-contingent ring in the corresponding eye, and observers centered both rings on the location of the target dot shown in the dominant eye. Figure 2 shows the horizontal positions of the dominant (red) and non-dominant (blue) eyes of 11 observers during 4.5 s of the alignment and eye dominance tasks. Figure 2A shows the mean data across observers and trials. The data show the mean horizontal position of the dominant eye (red) and non-dominant eye (blue), shading shows bootstrapped 95% confidence intervals. Figure 2B shows the data for each observer as the mean data across 40 trials.

The time interval marked by the dark gray shaded box indicates the time between the start of successful binocular fixation and the end of the ocular alignment task at 0 ms. The light gray shaded box from 0 to 300 ms indicates the interval corresponding to the eye dominance task. Data points before -1000 ms correspond to a period during which subjects were attempting to align their binocular gaze, data points after 300 ms correspond to free viewing, blinking and rest periods.

During the final 1 s of the alignment task (dark gray shaded region), the mean gaze direction of both eyes was within 0.5° of the location of the fixation target, and this gaze posture was maintained for the duration of the eye dominance stimulus (light gray shaded region). At the end of the trial, gaze alignment is more variable as feedback was removed and subjects were free to rest and blink. All eleven observers were able to complete this task successfully and no trials were aborted. These results are as expected for healthy, visually normal observers.

### Experiment 2: asymmetric convergence

**Figure 3:**
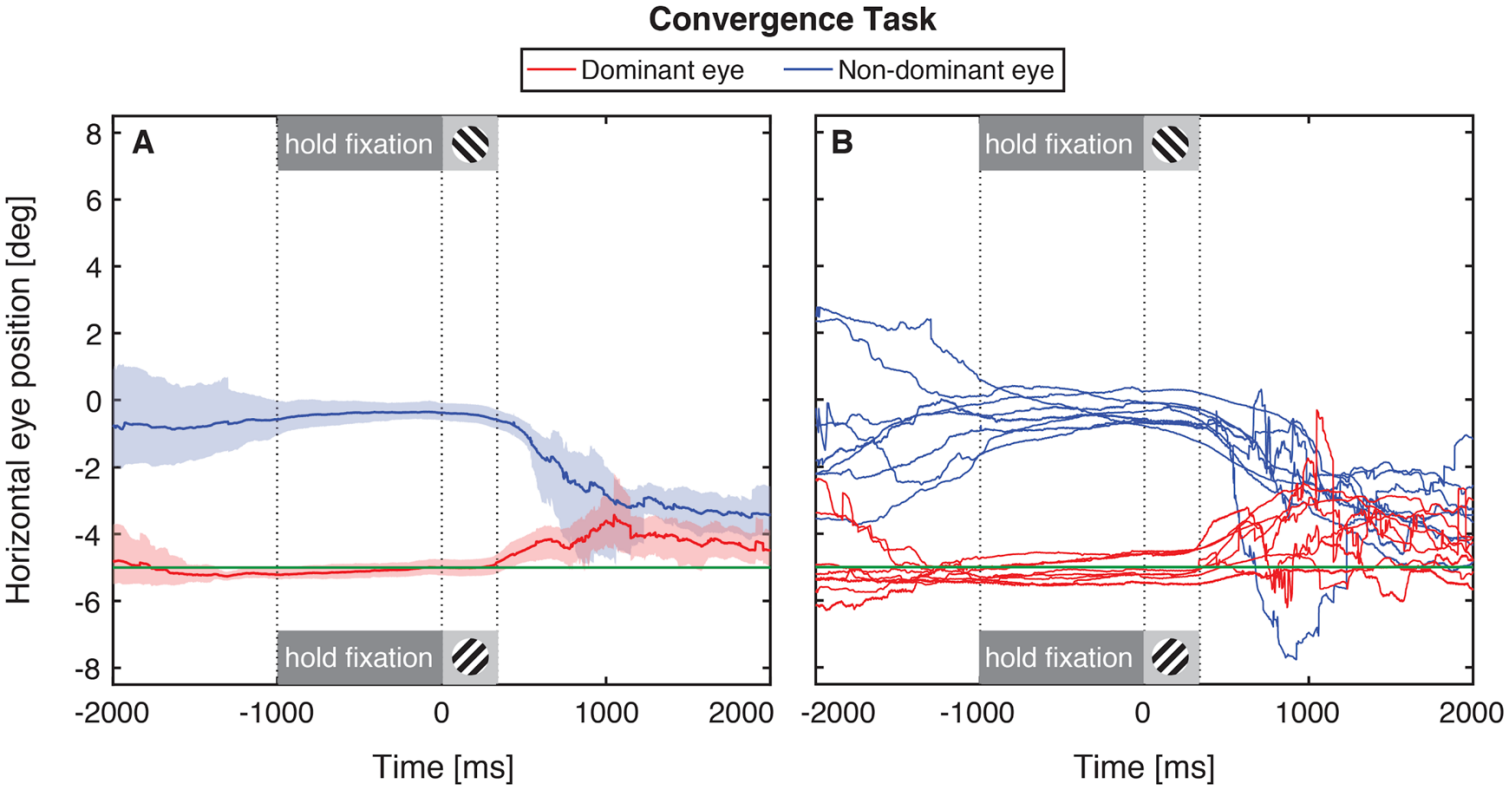
Horizontal gaze during convergent fixation. As Fig. 2, except the data are for 8 observers in the convergent fixation task in Experiment 2.

Observers were required to converge their non-dominant eye 5° (8.75Δ) nasally for at least 1 s before completing the eye dominance test. The results are plotted in Fig. 3 in the same format as those in Fig. 2 for Experiment 1. Eight of the eleven subjects were able to finish the experiment session. Three observers aborted the entire experiment, six observers aborted a subset of trials (by subject: 13, 4, 3, 2, 8 and 7) but were able to reach the end of the 40-trial session; and two observers successfully completed all trials without any aborted trials. Among the subjects who completed the experiment, the mean number of aborted trials was 4.6 (standard deviation 4.5).

The results are plotted as in Experiment 1, for eight observers. During the final 1 s of the alignment task (dark gray shaded region), the non-dominant eye (red curves) was close to the direction of the target (horizontal green line) and the non-dominant eye generated approximately 5° of asymmetric convergence for at least 1 s before the eye dominance test. Importantly, the convergence was maintained throughout the eye dominance test (light gray shaded region). At the end of the trial, after the removal of the monocular fixation target and binocular feedback, both eyes returned to binocular alignment within 1 s. Note that both eyes moved towards a position between the gaze directions of both eyes, suggesting that both eyes contributed partially to the convergence effort. This result shows that gaze-contingent visual feedback could be used to transiently induce asymmetric convergence in 73% of normally-sighted observers and that gaze realigned after the trial end.

### Experiment 3: asymmetric divergence

**Figure 4:**
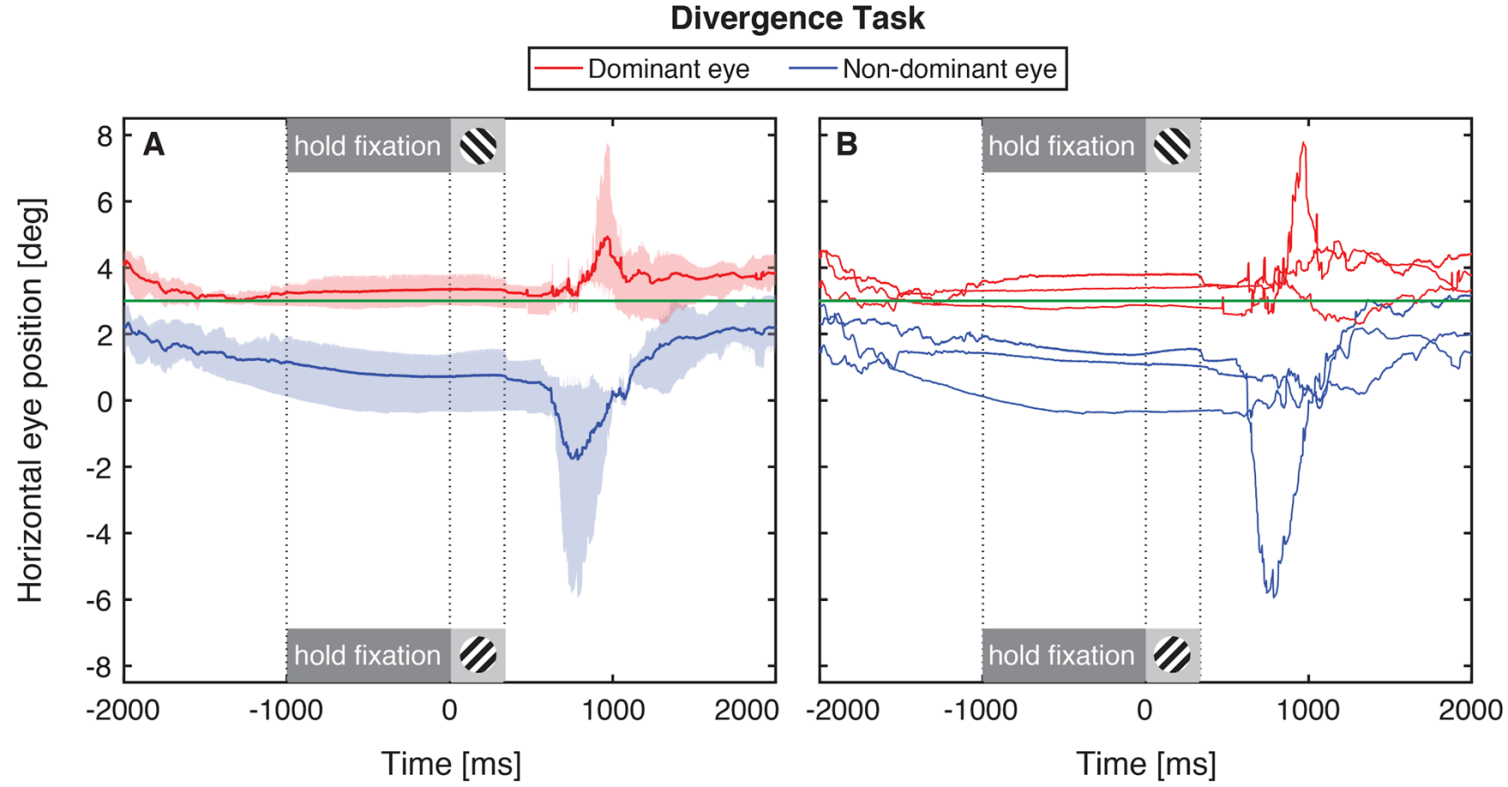
Horizontal gaze during divergent fixation. As Fig. 2, except the data are for 3 observers in the divergent fixation task in Experiment 3.

Observers were required to diverge their non-dominant eye 3° (5.24Δ) temporally for at least 1 s before completing the eye dominance test. The results are plotted in Fig. 4 in the same format as those in Figs. 2 and 3. Only three of eleven subjects were able to reach the end of the 40-trial session of this task. Eight observers aborted the entire experiment, two observers aborted a subset of trials (6 trials for one observer, 2 for the other) and one observer successfully completed all trials without any aborted trials. Of the subjects who completed the experiment session, the mean number of aborted trials was 2.7 (standard deviation 3.1).

The results are plotted as in Experiments 1 and 2 for the three reliable observers. During the final 1 s of the alignment task (dark gray shaded region), the dominant eye (red curves) was close to the direction of the target (horizontal green line) and the non-dominant eye generated approximately 3° of asymmetric divergence for at least 1 s before the eye dominance test. Importantly, the divergence was maintained throughout the eye dominance test (light gray shaded region). At the end of the trial, after the removal of the monocular fixation target and binocular feedback, one of the three observers showed a strong divergence response, but both eyes of all three observers moved towards a position between the gaze directions of both eyes, suggesting that both eyes contributed partially to the divergence effort. This result shows that gaze-contingent visual feedback could be used to transiently induce asymmetric divergence in 27% of normally-sighted observers. Two of the observers who were able to perform the divergence task were among the eight observers who were also able to complete the convergence task.

### Oculomotor learning

**Figure 5:**
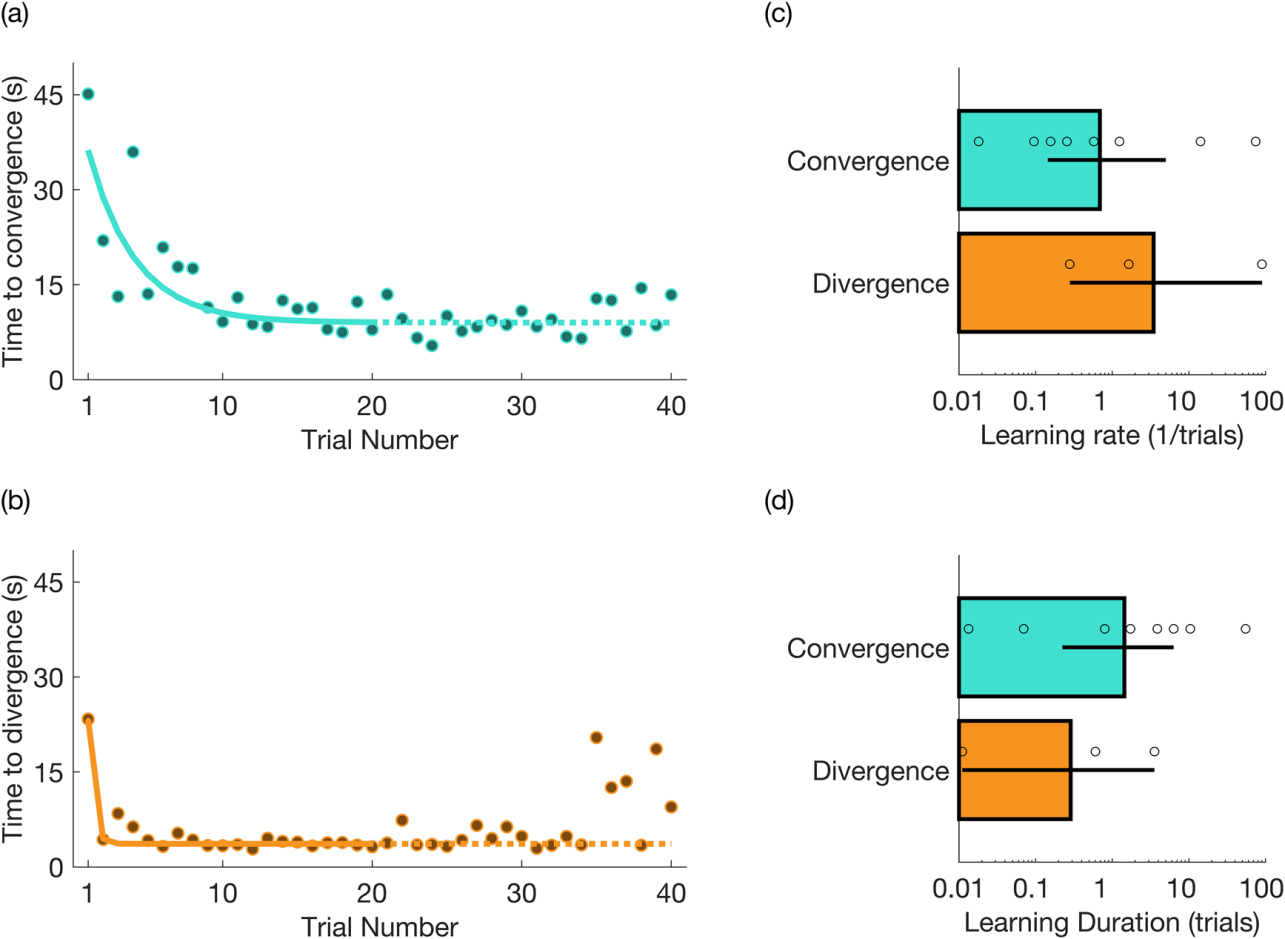
Oculomotor learning. The duration required to achieve target fixation (mis)alignment is shown across the 40 trials in each condition. Panels (**a**) and (**b**) show the results of a group analysis, in which the duration required to achieve misalignment was averaged across (**a**) 8 observers in Experiment 2, asymmetric convergence, and (**b**) 3 observers in Experiment 3, asymmetric divergence. The curves show the best fitting exponential decay functions. Panels (**c**) and (**d**) show the fit parameters, when trial durations are fit individually for each observer (see Figure S2 for individual plots). The bars in (**c**) and (**d**) show the learning rates (1/tau), and learning durations (tau), respectively, for the observers in each condition (see text for details). Error bars represent 95% bootstrapped confidence intervals.

To estimate whether there was any learning involved in performing the oculomotor alignment task, we examined the time from the start of each trial to the time at which observers maintained 1 s of trained alignment. Aborted trials were excluded from the analysis. Figure 5 shows the mean duration required to achieve target alignment on each trial for Experiments 2 and 3. Figure 5a shows the data averaged across the eight observers in the convergence task and Fig. 5b shows the average data for three observers in the divergence task. The curves show the best fitting three-term exponential decay function of the form:

$$T = N_{0} \times \exp\left( { - \frac{N - 1}{{\tau}}} \right) + c$$

where *T* is the duration of each trial, *N* is the trial number, *N*_*0*_ is the duration at the first trial, τ represents the learning duration (*1/τ* is the rate of learning) and parameter *c* controls the asymptote of learning. In addition to fitting the group-average data with an exponential decay function, we also fit the trial durations individually for each participant. The parameter estimates from these fits are shown in Fig. 5c,d, with scatter points representing individual observers (see Figure S2 in the Supplemental Materials for the individual plots, which are shown with and without the durations of previous aborted attempts added to the trial durations).

The results show that the time taken to obtain the target fixation pattern decreases rapidly over the first few trials, suggesting that ocular alignment is quickly learned by those observers who are able to perform this task. On average, the observed learning duration was 1.44 trials (95% CI [0.17 6.06]) in the Convergence condition, and 0.29 trials (95% CI [0.01 3.56]) in the Divergence condition, and was not significantly different for the each of the two observers who completed both conditions (p = 0.99 and p = 0.45 using individual permutation tests, see Figure S2 for details). We also note a slight increase in the time required to achieve misalignment in the last five trials of the divergence task, which appears to be driven by one observer (Figure S2), and may be related to fatigue^111^.

### Sensory eye dominance

Figure S3 in the Supplemental Materials shows the balance contrast across four conditions, a pretest condition that was used to quantify ocular dominance before any oculomotor training, and during the eye dominance tests in Experiments 1, 2 and 3. The balance contrast is the contrast of the grating presented to the non-dominant eye at which observers were equally likely to report the orientation of the grating in either eye. The results show the mean and 95% confidence intervals of the balance contrast for eleven observers in the pretest condition, eleven observers in Experiment 1, eight observers in Experiment 2 and three observers in Experiment 3. We note that there was somewhat larger variability in the balance contrast estimates in Experiments 1–3 compared to the initial pre-test. This variability could be due to the reduced number of trials during the experiments (40 trials) compared to the pretest (80 trials). Another source of variability could be the brief stimulus duration (300 ms in all tasks), which might reflect biases characteristic of the onset stage of binocular rivalry^112^. Despite this variability, we note that balance point estimates are nevertheless well-correlated within observers for the pre-test and the bifoveal task (r(9) = 0.61, p = 0.046).

A one-way repeated measures ANOVA with unequal sample sizes showed that there was no statistically significant effect of experimental condition (F_(3,19)_ = 0.047, p = 0.986). We therefore tentatively speculate that changes in ocular alignment transiently induced in normally-sighted observers may not immediately be associated with a change in sensory eye dominance. However, another possibility is that asymmetric vergence and convergence differentially change accommodation in the two eyes, which would in turn influence the measured balance contrast. Further studies measuring refraction before and after training would be necessary to differentiate between these possible interpretations.

## Discussion

Strabismus is a prevalent (3–5%) impairment of ocular alignment that is associated with a range of perceptual deficits and social disadvantages. Current methods to treat strabismus include surgical or optical alignment and vision therapy training. Estimates of the efficacy of each of these methods vary and there are well-documented risks of side effects and high levels of recidivism. The present study is an exploratory investigation in healthy observers of whether real-time visual feedback may be used to modify interocular alignment in healthy observers. The results of Experiment 2 show that 73% of healthy, naive observers were able to learn to converge their eyes in a few trials lasting several seconds (12.44 s ± 8.68 s; M ± SD; Fig. 5) to transiently simulate esotropia.

### Eye alignment in healthy and natural viewing

Eye alignment is characterized by a prominent binocular coordination, both at fixation and during eye movements. At fixation, the eyes show a posture that is unique at each gaze direction and vergence angle. According to the binocular extension of Listing’s Law^113^, the relative torsion of the eyes is meant to align the horizontal meridians, thus facilitating stereo correspondence. In addition, eye movement planning has been shown to be adapted to scene statistics^114,115^. In fact, vergence changes occur during saccades in order to bring the binocular fixation point to the most likely depth in the visual scene^114,116^. Effective and fine adaptation mechanisms are required to ensure a proper eye alignment against development, injuries and aging^56–60^. While these mechanisms are generally effective in healthy subjects, a residual functionality has been evidenced in subjects with binocular dysfunctions^50–54,100,117^. Our results identify plasticity in healthy adult observers that may possibly be engaged to possibly improve, rehabilitate or restore eye alignment in observers with vergence disorders.

### Eye alignment in binocular dysfunction

Standard vision therapy approaches do not record gaze direction and their outcomes are based on subjective reports of double vision. In contrast, the present feedback-based approach employs real-time measures of gaze direction from objective eye tracking. In future work, we propose to use this feedback-based method in an attempt to investigate whether real-time visual feedback can be used to change interocular alignment in patients with strabismus. In principle, manipulations of gaze direction opposite to those used here may be used to transiently simulate orthotropia in patients with strabismus, and preliminary data from one exotrope observer suggest that it can.

### Strengthening the effects of oculomotor training

The objective of the current study was to test whether it is at all possible for healthy participants to learn to voluntarily modify ocular alignment through dichoptic oculomotor training. In order to translate this approach to patient populations, the dichoptic training procedure could be potentially enhanced in several ways. For instance, we employed naïve observers who volunteered for up to an hour in return for course credit and therefore did not examine whether longer periods of training could have led to higher success rates. In pilot studies, we decreased the angle of deviation for asymmetric divergence to 3° (5.24Δ) because the task was more difficult than training 5° (8.75Δ) of convergence even for experienced observers. In recent work, we have examined the transfer of learning from smaller angles of convergence or divergence^118^, and we demonstrate that stepwise incremental training can be used to obtain progressively larger divergence and convergence angles. We also show that such oculomotor learning is partially retained over a period of at least one week (Figure S4). Further studies on the retention of oculomotor training would be necessary to establish the utility of this method for clinical applications.

Additionally, our 3D display setup likely caused vergence-accommodation conflicts, which are known to hinder binocular fusion^111^, and might have reduced the training efficacy. Employing simulated peripheral dioptric blur in richer, naturalistic training environments may help counteract the adverse effects of vergence-accommodation conflicts^102^. Refractive error is also linked to stronger vergence-accommodation conflicts^119^, which may have implications particularly for anisometropic strabismic patients who may have different refraction in the two eyes. In recent work however we have demonstrated a synergistic link between eye and hand movements in 3D, which persists even with simulated asymmetric visual impairment^103^. It might therefore be possible to exploit this link via eye-hand coordination tasks^120^ to enhance the training effects we find here.

### Eye dominance

The sensory eye dominance task was designed to estimate changes in binocularity during training. Given that the misaligned target was presented to the non-dominant eye, we might expect greater attentional task demands in this eye, and thus a shift in eye dominance towards the non-dominant eye^121^. This could potentially provide a mechanism to strengthen the weaker eye in amblyopic and strabismic patients. The results of the sensory eye dominance measurement show that ocular misalignment might not immediately change the level of eye dominance in healthy observers. However, as we note in the results, changes in accommodation could significantly impact these measures. As we did not measure refraction before and after training because all observers were healthy young adults who wore their best current refraction, these results are inconclusive, and further tests in a larger number of healthy subjects with estimates of refractive error would be required to establish the effect of this oculomotor training on perceptual function. We also note that previous work has demonstrated mixed results in this area. For example, surgical changes in ocular alignment do not immediately lead to changes in perceptual function^61–63^, whereas other groups have reported immediate benefits in stereoacuity following ocular alignment surgery^51–55^. In either case, the present measurements of eye dominance were obtained in normally-sighted observers who do not experience strong suppression, and it is possible that the results will be different in strabismic observers who experience chronic or transient suppression. It is also possible that longer periods of training may be required to observe sensory changes.

## Conclusion

The present paper is a proof of concept study to examine the effectiveness of real-time visual feedback to quantify and simulate convergent and divergent strabismus with self-initiated asymmetric vergence movements. The results are a promising demonstration that dichoptic feedback may be used to transiently simulate esotropia and exotropia in normally-sighted healthy observers. Combining perceptual learning with visual feedback could produce improvement of the eye mobility and visual perception. Future work is necessary to test whether this method is effective in patients with strabismus, but the objective is that this approach may be used to augment and maintain other interventions.

## Supplementary information

Supplementary Figures

## Data Availability

The datasets for the current study are available in the following OSF repository:  https://osf.io/h7b2e/.
