# Supplementary material for: A dichoptic feedback-based oculomotor training method to manipulate interocular alignment
Source: Sci Rep. 2020 Sep 24;10:15634. doi: 10.1038/s41598-020-72561-y (PMC7515870; doi:10.1038/s41598-020-72561-y)
Supplement: Supplementary file 1 — Supplementary Figures [file 41598_2020_72561_MOESM1_ESM.docx]

**Supplemental Materials:**

A Dichoptic Feedback-Based Oculomotor Training Method to Manipulate Interocular Alignment

Andrea Caoli, Silvio Sabatini, Agostino Gibaldi, Guido Maiello, Anna Kosovicheva, Peter Bex


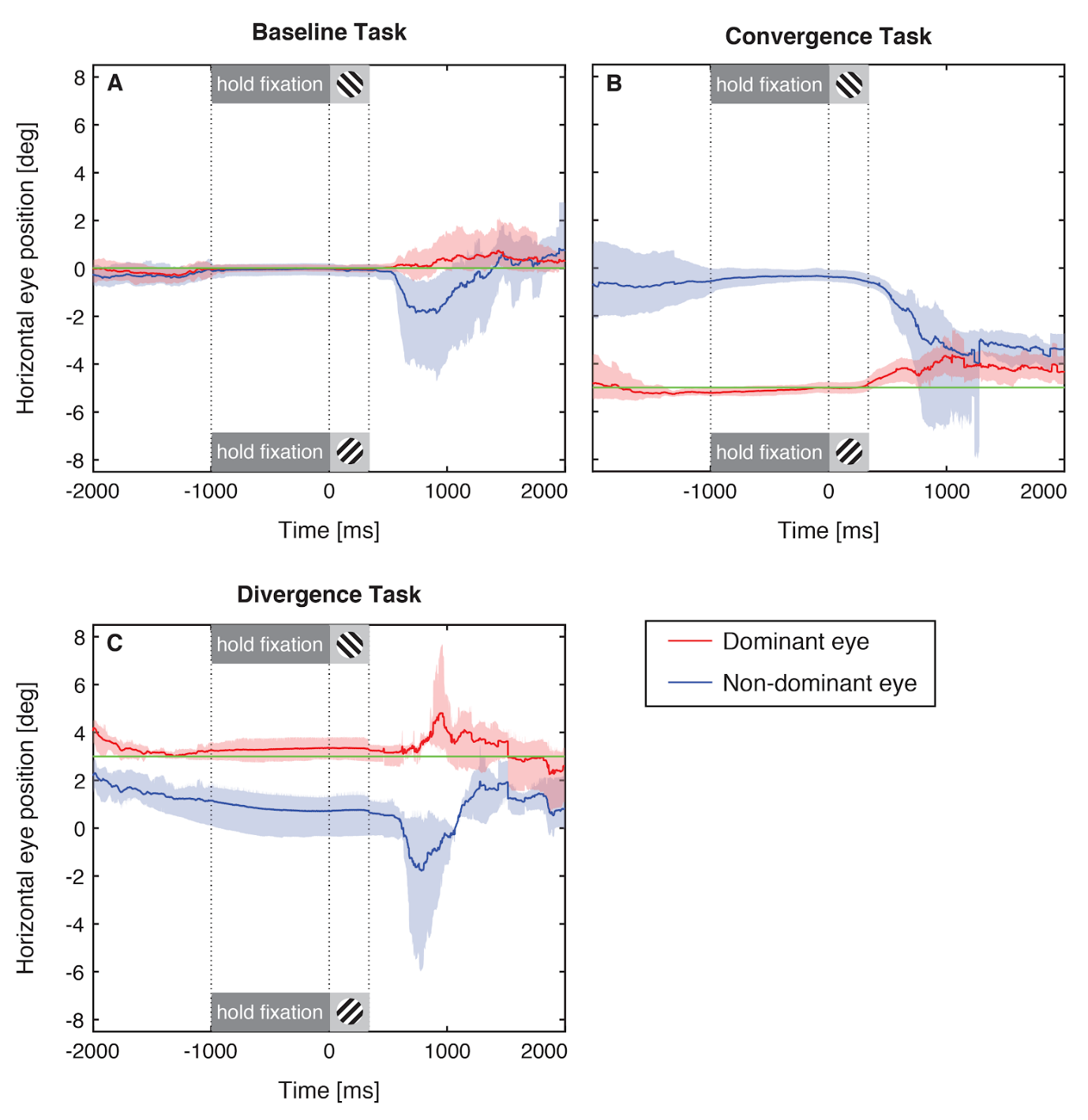


**Figure S1. Horizontal gaze position with temporal overlap removed.** On some trials, observers were able to complete the task and initiate the next trial quickly, which resulted in a small degree of temporal overlap in the gaze position traces analyzed across trials. The horizontal gaze positions are plotted for the (A) Bifoveal, (B) Convergence, and (C) Divergence tasks, removing time points from the end of each trial in which there was any temporal overlap (similar to Figures 2A, 3A, and 4A; see figure captions for details).


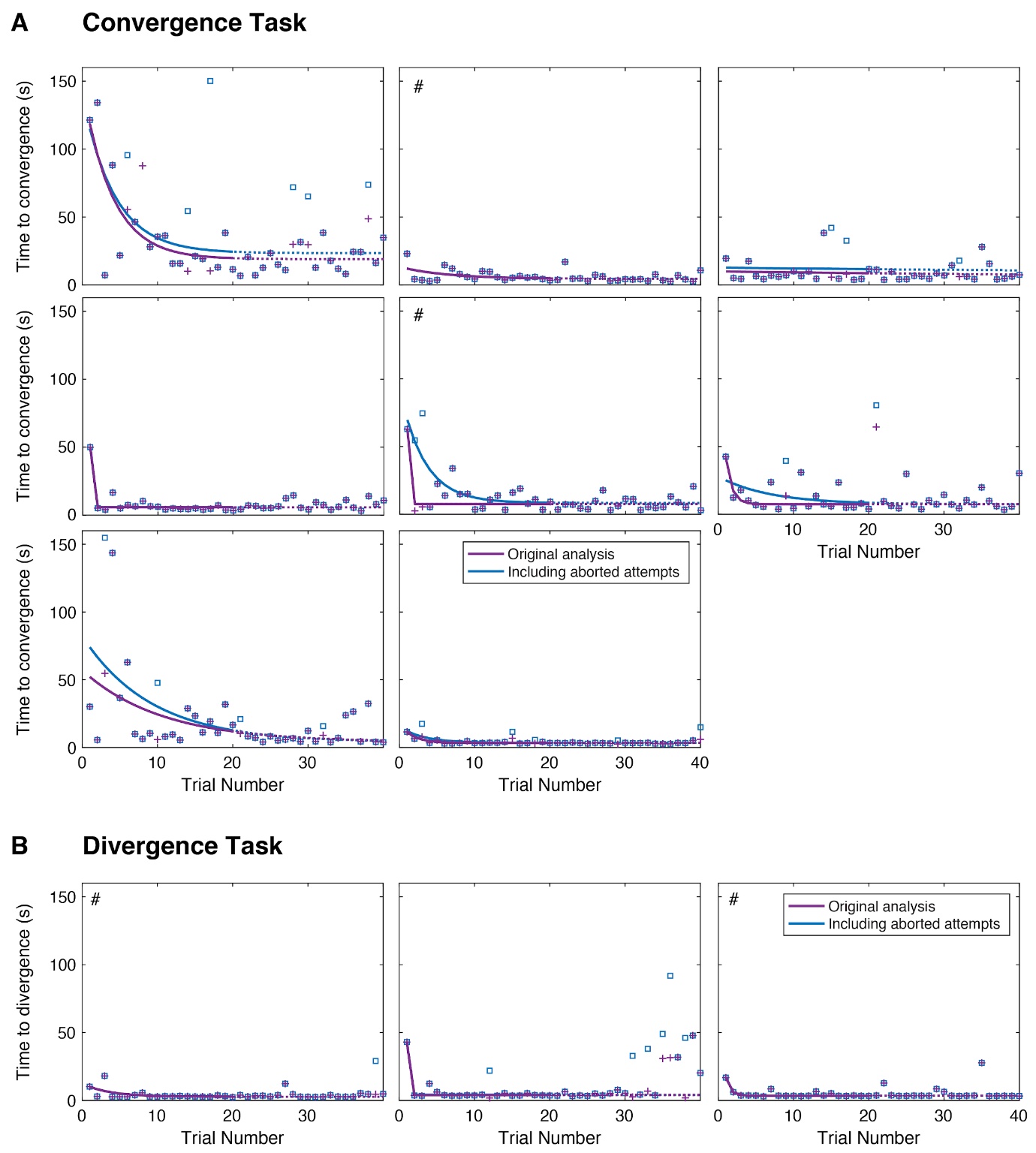
**Figure S2. Oculomotor Learning: Individual Participant Fits.** The time required to attain target fixation misalignment was calculated for individual participants and fit with a three-term exponential decay function for each participant in the (A) Convergence and (B) Divergence tasks (see text and Figure 5 for description). The purple curves (and plus symbols) show the fits excluding the duration of previous aborted attempts, and the blue curves (and open squares) show the fits with the duration of previous aborted attempts added to the total duration. Note that in the event of consecutive aborted attempts, multiple trial durations were summed together. One outlier data point was removed from the first panel of (A), which had a duration of 570 s (only when aborted attempts were included). Observers who successfully completed both the convergence and divergence tasks are indicated by the # symbol.

To compare the learning durations between Convergence and Divergence conditions, we performed separate permutation tests for the two observers who were able to complete both tasks. On each iteration, the condition labels (Convergence and Divergence) for matched pairs of trials were randomly shuffled between the two conditions (i.e., pairwise scrambling for trial 1, 2, etc.), and the data were re-fit with an exponential decay function. This process was repeated for 1,000 iterations to produce a null distribution of differences in learning duration, and *p*-values were calculated from the proportion of observations in the null distribution that were more extreme than the observed difference between conditions (two-tailed test). The difference in learning duration was not significant for either observer (*p* = 0.99 and *p* = 0.45).


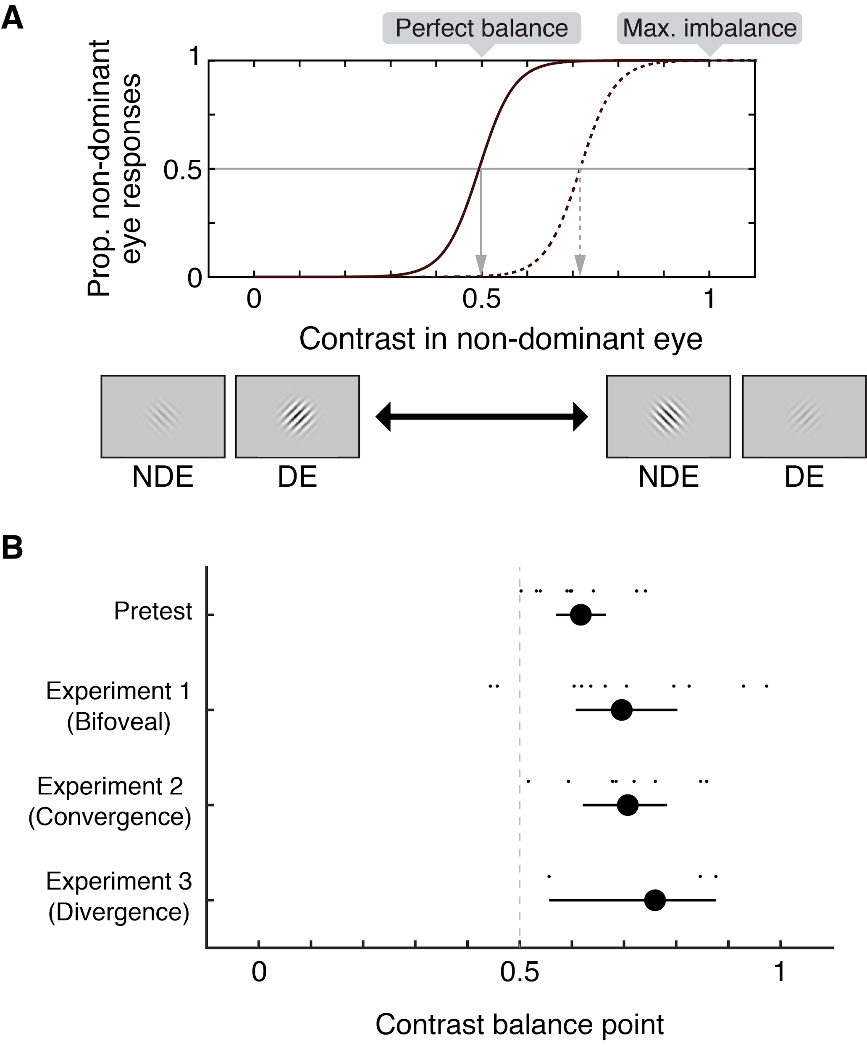


**Figure S3**. **Sensory Eye Dominance.** (A) Responses from the eye dominance task were used to estimate the balance contrast– the contrast of the grating presented to the non-dominant eye so that the orientation of the grating in either eye is reported with equal probability (NDE = non-dominant eye, DE = dominant eye; see Methods for details). Contrast levels were varied with a QUEST staircase procedure, and the balance contrast was estimated from the point of subjective equality (PSE). Contrast levels are plotted relative to the non-dominant eye (established during the pretest), such that a value of 0.5 indicates perfect balance (i.e., equal sensory eye dominance; solid line), and values above 0.5 indicate greater levels of imbalance (dashed line), up to a value of 1.0 (maximum imbalance). In other words, values above 0.5 indicate that the contrast level must be larger in the non-dominant eye to produce perceptual reports of equal frequency in the two eyes (B) Balance contrast for four conditions; a pretest (baseline) condition (N=11 observers) and Experiments 1 (N=11), 2 (N=8) and 3 (N=3). Data points show the mean and bootstrapped 95% confidence intervals.


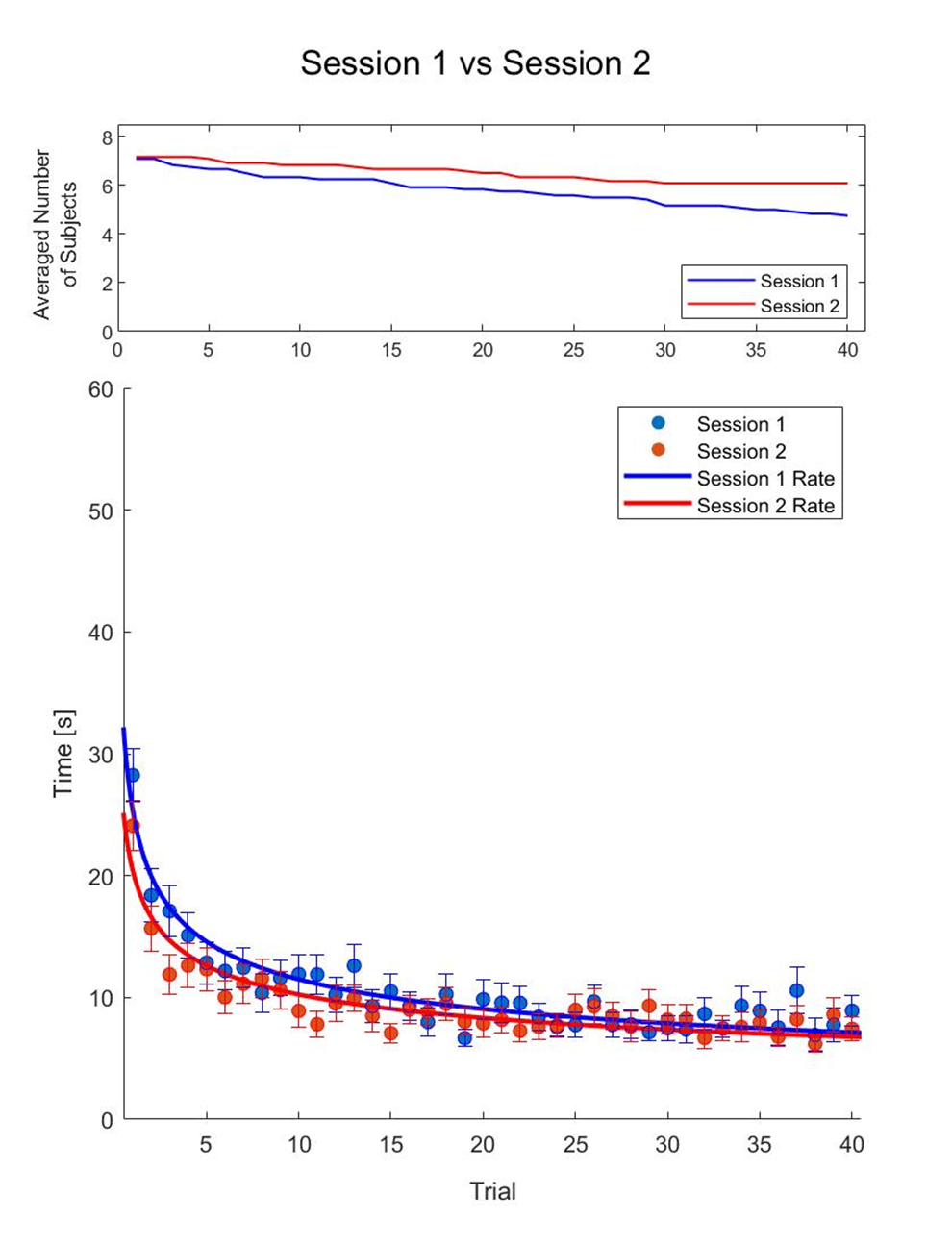


**Figure S4. Retention of Oculomotor Training.** Results from a study with a separate group of participants (N=32; Walter, Taveras Cruz, & Bex, 2020), comparing the time taken to achieve the target posture across two sessions (lower panel) that were completed one week apart (first session shown in blue, second session in red). Results are averaged over conditions with variable training directions and magnitudes (2º and 4º of divergence and convergence). The recorded time was significantly shorter in Session 2 compared to Session 1 (*t* = –2.695, *p* = 0.007). As in the present study, observers were allowed to abort trials in which they could not achieve the target posture. The upper panel shows the number of observers able to complete each trial, averaged across four conditions with 8 participants each. The number of trials completed was significantly larger in Session 2 compared to Session 1 (*t* = 2.134, *p* = 0.033). (Figure reproduced with permission from Walter, Taveras Cruz, & Bex, 2020 ^1^ and licensed under a [CC BY-NC-ND 4.0](https://creativecommons.org/licenses/by-nc-nd/4.0/) license).

**REFERENCES**

1. Walter, K., Taveras-Cruz, Y. & Bex, P. Transfer and retention of oculomotor alignment rehabilitation training. *J. Vis.* **20**, 9–9 (2020).
